# Ventricular fibrillation induced by fever in structurally normal hearts

**DOI:** 10.3389/fcvm.2023.1230295

**Published:** 2023-09-25

**Authors:** Ji-Fang Ma, You Zhou, Hai-Xia Fu

**Affiliations:** Department of Cardiology, Fuwai Central China Cardiovascular Hospital, Zhengzhou, China

**Keywords:** ventricular fibrillation, fever, brugada syndrome, long QT syndrome, idiopathic ventricular fibrillation

## Abstract

Ventricular fibrillation (VF) is a life-threatening arrhythmia that usually happens in patients with structural heart diseases. However, fever-induced ventricular fibrillation in structurally normal hearts was reported, and the four main diseases associated with these cases were Brugada syndrome, long QT syndrome, idiopathic ventricular fibrillation, and non-cardiovascular diseases. In this review, we analyzed this phenomenon and its clinical characteristics.

## 1. Introduction

Ventricular fibrillation (VF), an emergency condition often precipitating sudden cardiac death in patients with structural heart disease, is often triggered by electrolyte imbalance, hypoxemia, and drug-related toxicity. However, it is occasionally induced by fever in structurally normal hearts.

Sporadic cases have been reported. Most of them focused on Brugada Syndrome (1–11). Fever can trigger the Brugada-type electrocardiogram (ECG) and increase the propensity for VF in structurally normal hearts. Furthermore, Long QT Syndrome type 2 (LQTS-2) (12, 13) and idiopathic VF storm (14, 15) can also trigger VF during fever. In some patients, an implantable cardioverter-defibrillator (ICD) pacemaker was needed. In some patients with idiopathic VF, cardiac electrophysiological examination and catheter ablation might be a promising choice.

This review aims to demonstrate the clinical characteristics of patients presenting with ventricular fibrillation induced by fever without structural heart diseases (Table 1).

**Table 1:** The characteristics of patients with ventricular fibrillation during fever.

	Brugada syndrome	LQTS-2	Idiopathic VF
Total patients	23	3	5
Male (*n*,%)	17/20 (85%)	3/3 (100%)	2/5 (40%)
Age (y)	41.2 ± 11.42	45 ± 21.28	62.4 ± 9.34
	(21–56)	(26–68)	(51–75)
History of syncope (*n*, %)	4/20 (20%)	2/3 (66.67%)	5/5 (100%)
Family of SCD (*n*, %)	6/20 (30%)	0	0
Temperature at fever (°C)	38.3–40	38.5–39.7	38.5–39
Infection cause fever (*n*)	Pneumonia (7), bronchitis (1), influenza-like (2), gastroenteritis (1), appendicitis (1), urinary tract infection (1)#	Pneumonia (1), upper respiratory tract (1), urinary tract infection (1)	Pneumonia (3), upper respiratory tract (1), influenza-like (1)
Bacteriological testing in blood	Streptococcus (Sato 2018)	-	Escherichia coli (Pasquie 2004)
Positive EPS (*n*, %)	4/20 (20%)	-	3/5 (60%)
ICD (*n*, %)	5/20 (25%)	3/3 (100%)	4/5 (80%)
References	(1–11)	(12, 13)	(14–15, 25)

LQTS, long QT syndrome; VF, ventricular fibrillation; SCD, sudden cardiac death; EPS, electrophysiology study; ICD, implantable cardioverter-defibrillator.

## 2. Diseases associated with VF induced by fever

Brugada syndrome, LQTS-2, and idiopathic ventricular fibrillation are three main diseases that combine with ventricular fibrillation during fever. In addition, some non-cardiovascular diseases can also lead to ventricular fibrillation during fever. Details of different diseases were described as follows.

## 3. Brugada syndrome (BrS)

Brugada Syndrome is a special disease characterized by a coved ST segment elevation in precordial leads (V1–V3). It happens nine times more frequently in men (16). BrS often combines with severe ventricular arrhythmia, which triggers sudden cardiac death in young men with structurally normal hearts (16, 17).

Fever-induced ventricular fibrillation in BrS is common (1, 2, 3, 4, 5, 6, 7, 10, 11). The average temperature of fever was high (38.3–40°C), with pneumonia as the most common precipitating factor. Other reported factors, including bronchitis, influenza, gastroenteritis, appendicitis, and urinary tract infection, can induce fever and ventricular fibrillation in patients with BrS. A multicenter study (SABRUS) (9) described the phenomenon of fever-related arrhythmic events in 678 patients with BrS. A high incidence, 6% of patients, experienced fever-related arrhythmic events: 80% were men and the average age was 29 ± 24 years. Among them, 80% of patients presented with cardiac arrest, 40% of patients had a history of syncope, and 17% of patients experienced arrhythmic storm. Younger patients (age < 16 years) had a higher rate, of 65%, of febrile illness-related arrhythmia.

Brugada-ECG [a coved ST segment elevation in precordial leads (V1–V3)] is a prominent indication to recognize those patients with BrS during fever. Nearly 66% of patients presented spontaneous type 1 Brugada-ECG during fever (9). Adler et al. (7) studied 1,311 patients to recognize the relationship between fever and Brugada-ECG. They concluded that the type 1 Brugada pattern appeared 20 times more in the febrile group than in the afebrile group (2% vs. 0.1%, respectively, *P*  =  .0001). However, the fever-induced ECG pattern was no longer sustained when the fever subsided. Ozben (18) and Yalin (6) also reported on young male patients who presented with ST-elevation in the right precordial during fever, which then disappeared after the fever resolved. In those patients without Brugada-ECG, the Ajmaline test might be a good choice to induce type 1 Brugada-ECG. Besides, the presence of S-wave in lead 1 suggested a conduction delay localized in the right ventricular outflow tract (RVOT), which was a possible underlying arrhythmogenic substrate to recognize patients with BrS (19).

Mutations of the sodium-channel gene (*SCN5A*) were associated with BrS. Mutations of *SCN5A* may inhibit the sodium channel current and cause heterogeneous loss of action potential dome during the plateau phase 2 in the right ventricular epicardium (20–22), producing a Brugada-ECG — coved ST segment elevation in precordial leads (V1-V3). The temperature-related Brugada-ECG change might be related to the ionic shifts. Keller et al. (23) reported on a Brugada syndrome patient with fever-induced ventricular fibrillation. They found a mutation F1344S of SCN5A with a loss of sodium channel function. Dumaine R et al. (23) demonstrated an accelerated inactivation of the cardiac sodium channel at high temperatures. They found the Thr1620Met missense mutation of the sodium-channel gene (SCN5A) had faster decay and longer recovery time during the early phases of the right ventricular action potential than the wild type at 32°C, suggesting a varied cardiac sodium channel function dependent on different temperatures. This might be the main reason for BrS patients with high rates of ventricular arrhythmia during fever.

Fever-induced Brugada-ECG pattern is associated with a high risk of ventricular fibrillation and sudden cardiac death. Mizusawa et al. (8) found that fever-induced type 1 Brs ECG pattern had a high risk of arrhythmic events. In these patients, the cardiac electrophysiological examination (EPS) is quite necessary to evaluate the risk of sudden cardiac death. Patients who had positive results of ventricular arrhythmia by EPS were often recommended implantable cardioverter-defibrillator (ICD) pacemaker, which might decrease the risk of sudden cardiac death.

Therefore, fever might trigger Brugada-ECG presentations and increase the risk of sudden cardiac death in generally healthy patients. Patients with known fever-induced VF should be kept under serious surveillance during fever and started on antipyretic therapy as soon as possible (24).

## 4. Long QT syndrome type 2 (LQTS-2)

LQTS is a genetic channelopathy associated with life-threatening arrhythmia that can be triggered by fever (25), especially type 2 long QT syndrome of go-go-related gene (*HERG*) mutation. The temperature during fever ranged from 38.5 to 39.7°C. Pneumonia, upper respiratory tract infection, and urinary tract infection were the main causes of fever. The average QTc interval in most patients was normal at baseline. However, the normal QTc interval was significantly prolonged in all patients during fever.

Lim et al. (13) reported on a young man with fever-induced polymorphic ventricular tachycardia and QT interval prolongation. The QTc interval was prolonged from 365 ms to 535 ms during fever. The QTc returned to normal 1 h later after the resolution of the fever. Amin et al. (12) identified a mutation of A558P in the *HERG* gene that encodes the α subunit for the delayed rectifier K current (*I* kr) in a patient with fever-induced QT interval prolongation. The HERG channel function is often temperature-dependent. High temperature (up to 40°C) prolonged the action potential and triggered the early after-depolarizations (EADs) that were associated with the initiation of torsades de points. ICD implantation was recommended in those patients.

## 5. Idiopathic ventricular fibrillation

Patients diagnosed with idiopathic ventricular fibrillation during fever in the literature ranged from 51 to 75 years old. Some of them had a history of syncope. They all presented with high fever (38.5–39°C). Pneumonia, respiratory tract infection, or infecting influenza was the main cause of fever.

Pasquie et al. (14) observed three patients without structural heart disease or repolarization abnormalities with fever as the only factor associated with VF. The electrophysiological mapping of one patient found the origin of the trigger located at the Purkinje arborization of the anterior wall of the right ventricle. He remained free of VF after a follow-up of 18 months.

Omari et al. (15) reported on a 75-year-old patient who experienced ventricular tachycardia and ventricular fibrillation at a high fever of 39°C. The ventricular arrhythmia disappeared when the patient returned to a normal body temperature. Furthermore, the following electrophysiological study failed to induce any ventricular arrhythmias.

Arias MA (26) reported on a 51-year-old woman with frequent premature ventricular complexes (PVCs) and syncope at 101.3°F with otherwise normal vital signs. The PVCs had frequent short coupling intervals (230 ms) with left bundle branch block morphology and a left superior frontal plane axis with a negative concordance pattern in precordial leads. The subsequent VF episodes were triggered by the same PVC morphology. The electrophysiological study found that a site at the apical free wall of the right ventricular with preceding Purkinje potentials was the earliest ventricular activation site. Catheter ablation at this point completely abolished the PVCs. The patient remained free of any ventricular tachycardia during the 7-year follow-up.

All of the origin of the trigger was located at the Purkinje arborization of the anterior wall of the right ventricle. Catheter ablation of this target was a good choice to treat this idiopathic ventricular fibrillation. ICD pacemaker might be unnecessary in those patients accepting successful catheter ablation. However, a longer follow-up of the prognosis was needed.

## 6. Non-cardiovascular diseases

The other patients of fever-induced VF were distributed in carcinoma (27), APS-2 (28), and Andersen-Tawil syndrome (29). They all had structurally normal hearts. Ventricular fibrillation was manifested as the concomitant symptom. Patients also expressed characteristics of the primary disease.

Carcinoma was associated with fever-induced ventricular fibrillation. Fukuda et al. (27) reported on a 62-year-old man with papillary thyroid carcinoma who died of ventricular fibrillation while presenting a slight fever. The autopsy found the tumor occupied nearly the whole right ventricular cavity expanding toward the main trunk of the pulmonary artery.

Wang et al. (28) reported on a case of autoimmune polyglandular syndrome type 2 (APS-2). The patient was diagnosed with acute gastroenteritis during fever and fell into ventricular fibrillation and shock subsequently. The patient improved after the supplementation with hydrocortisone and fludrocortisone.

Maffe et al. (29) presented a patient diagnosed with Andersen-Tawil Syndrome (ATS) with cardiac involvement ranging from ventricular fibrillation to sudden death. The episodes of bidirectional ventricular tachycardia were identified during fever. An ICD was implanted.

Recently, coronavirus disease 2019 (COVID-19) pneumonia (30) was reported to be associated with a ventricular tachycardia storm which needed substrate-based catheter ablation, indicating an increased risk of electrical storm in patients with COVID-19 infection.

Therapy of the primary diseases might be helpful to control the fever-induced VF, including carcinoma, APS-2, ATS, COVID-19, and so on.

## 7. Limitations

Most studies associated with fever-induced VF were case reports, which varied in clinical assessment, prognosis, gene test, and therapy. This may increase the difficulties in consistency evaluation. Besides, the average follow-up of these studies was shorter than 2 years. The longer prognosis of such patients is unknown. Whether these patients presented again with recurring ventricular fibrillation during fever remains unclear. More studies are needed.

## 8. Conclusion

Fever-induced ventricular fibrillation in structurally normal hearts is not as rare as we presumed. It can happen in Brugada syndrome, Long QT syndrome type 2, idiopathic ventricular fibrillation, and non-cardiovascular diseases. The elevation of body temperature might disrupt the ionic current migration of cardiomyocytes and induce the heterogeneity of transmural dispersion. Then, what looked like a “normal” heart will be more prone to presenting ventricular fibrillation. ICD implantation might be important in LQTS and idiopathic ventricular fibrillation, and be optional in patients with BrS. Catheter ablation of the origin of the trigger has gained promising effects in idiopathic ventricular fibrillation.
